# Protein kinase D displays intrinsic Tyr autophosphorylation activity: insights into mechanism and regulation

**DOI:** 10.1002/1873-3468.13171

**Published:** 2018-07-23

**Authors:** Mathias Cobbaut, Rita Derua, Peter J. Parker, Etienne Waelkens, Veerle Janssens, Johan Van Lint

**Affiliations:** ^1^ Laboratory of Protein Phosphorylation and Proteomics Department of Cellular and Molecular Medicine Faculty of Medicine KU Leuven Belgium; ^2^ Leuven Cancer Institute (LKI) KU Leuven Belgium; ^3^ Protein Phosphorylation Lab The Francis Crick Institute London UK; ^4^ School of Cancer and Pharmaceutical Sciences King's College London UK; ^5^ Present address: Protein Phosphorylation Lab The Francis Crick Institute London UK

**Keywords:** autophosphorylation, dual‐specificity, kinase, protein kinase D

## Abstract

The protein kinase D (PKD) family is regulated through multi‐site phosphorylation, including autophosphorylation. For example, PKD displays *in vivo* autophosphorylation on Ser‐742 (and Ser‐738 *in vitro*) in the activation loop and Ser‐910 in the C‐tail (hPKD1 numbering). In this paper, we describe the surprising observation that PKD also displays *in vitro* autocatalytic activity towards a Tyr residue in the P + 1 loop of the activation segment. We define the molecular determinants for this unusual activity and identify a Cys residue (C705 in PKD1) in the catalytic loop as of utmost importance. In cells, PKD Tyr autophosphorylation is suppressed through the association of an inhibitory factor. Our findings provide important novel insights into PKD (auto)regulation.

## Abbreviations

**CBB;** Coomassie Brilliant Blue; DAG, diacylglycerol

**DMEM,**Dulbecco's modified eagle medium

**KD,** kinase‐dead

**PEI,** polyethyleneimine

**PH,** pleckstrin homology

**PKD,** protein kinase D

**RTK,** receptor tyrosine kinase

**SUVs,** small unilamellar vesicles

Protein kinases are essential for cellular life. They play a role in almost all physiological processes and their dysfunction is associated with a plethora of diseases 1. The activity and function of many kinases is regulated through phosphorylation, in particular through phosphorylation of the activation segment 2. This is especially the case for so‐called ‘RD’ kinases, which contain an arginine in the highly conserved HRD motif in the catalytic loop. The Arg residue in this motif creates a basic pocket together with a Lys residue located C‐terminally of the DFG motif that accommodates the phosphate group of the phospho‐Ser/Thr residue in the activation loop 3. In many cases, phosphorylation of activation segment residue(s) is dependent on the activity of an upstream kinase, but many kinases also possess an inherent autophosphorylation activity, which is physiologically relevant in many cases 4.

One of the most obvious examples of an activating autophosphorylation is found within the Receptor Tyrosine Kinase (RTK) group, where ligand‐induced dimerization results in *trans*‐autophosphorylation of the activation loop residues, and in generation of docking motifs to initiate the signaling cascade 5. However, kinase autophosphorylation extends beyond RTKs and is actually much more common than hitherto believed. In a recent comparative literature search of all RD‐kinases, it was found that about 63% have autophosphorylation capabilities and 45% autophosphorylate their regulatory activation loop residue(s) 4.

Kinases can be divided by their specificity toward their targeted residues. The majority of kinases are those that create phospho‐ester bonds on hydroxyl groups, and can be subdivided in Ser/Thr and Tyr kinases 6. For some kinases, however, such a distinction cannot be made so clearly. For example, several predominantly Ser/Thr kinases have been shown to also (auto)phosphorylate Tyr residues 7, 8, 9, 10, 11, 12, 13, 14, 15, 16, 17, 18, 19. The inverse, a Tyr kinase that also (auto)phosphorylates on Ser/Thr residues, is less common, but occurring nonetheless 20, 21. The role of this ‘dual specificity’ is well‐described for some kinases 9, 10, 18, 19. Other (auto)phosphorylation events are less well understood in terms of function, and sometimes dual specificity is only seen under specific circumstances 19. While it is known that some kinases have a dual specificity, the molecular determinants in kinases that confer to this property have not been examined in close detail.

The protein kinase D (PKD) family consists of three closely related isoforms in humans, and is part of the CAMK group 22. They play a versatile role in (disease) biology, ranging from migration to secretion, proliferation and invasion (for a recent review see 23). They consist of a large N‐terminal domain, followed by the kinase domain. The N‐terminal regulatory region encompasses an alanine/proline rich region (AP region), a tandem C1 domain binding diacylglycerol (DAG) and phorbol esters, an oligomerization domain, and a pleckstrin homology (PH) domain 24, 25, 26, 27, 28. Deletion mutagenesis approaches have indicated that the C1 and PH domains have an autoinhibitory effect on kinase activity, which is alleviated by activation loop Ser‐738/742 phosphorylation (hPKD1 numbering) 24, 25, 26. These phosphorylations are exerted by several PKCs, but PKDs can also autophosphorylate on Ser‐738 and Ser‐742 *in vitro*, and on Ser‐742 in cells 29, 30. Furthermore, PKD1/2 autophosphorylates *in vivo* at a C‐terminal Ser residue (Ser‐910 in PKD1). This phosphorylation is not contributing to PKD activation or maximal activity, but rather regulates the duration of PKD signaling in a cellular context 31.

Protein kinase D are also regulated through Tyr phosphorylation, for example in oxidative stress conditions. Upon exposure to H_2_O_2_, Tyr‐95 and Tyr‐463 in PKD1 are phosphorylated by Src and Abl respectively 32, 33, 34, 35. In PKD2 an additional Tyr residue is phosphorylated by Abl in oxidative stress at the P + 1 loop of the activation segment, causing an increase in substrate turnover 36.

In this paper, we describe that the PKD family members also display *auto*phosphorylation of this Tyr residue in the P + 1 loop, and we identify the molecular determinants for this unusual activity.

## Materials and methods

### Cell culture, antibodies and chemicals

HEK293 cells were grown in Dulbecco's modified eagle medium (DMEM) supplemented with 10% (v/v) Fetal Bovine serum (GE Healthcare, Little Chalfont, UK), 2 mm glutaMAX (ThermoFisher Scientific, Waltham, MA, USA), 100U·mL^−1^ Penicillin and 100 μg·mL^−1^ Streptomycin (ThermoFisher Scientific). Anti‐GST, Anti‐FLAG M2 antibody, HA antibody and agarose resins were purchased from Sigma (St. Louis, MO, USA). Glutathione sepharose 4B beads were from GE healthcare. Anti‐phosphotyrosine antibody (4G10) was from Millipore (Billerica, MA, USA), PKD anti‐pSer‐744/748 antibody, anti‐PKCδ antibody, secondary HRP‐linked goat anti‐Rabbit and Horse, anti‐Mouse antibodies were from Cell Signaling Technologies (Beverly, MA, USA). An in‐house site‐specific phospho‐Tyr antibody targeting pTyr in the P + 1 loop (CPApYLAPEV), which is cross‐reactive between PKD isoforms due to 100% homology of the epitope is described previously 36. Phorbol 12,13‐dibutyrate (PDB), ATP, STI‐571, PP2, CRT 0066101, CID 755673 and Hydrogen peroxide 30% (v/v) were from Sigma, Polyethyleneimine (PEI) was from Polysciences Inc. (Warrington, PA, USA).

### Oligonucleotides, plasmids and cloning

Plasmids encoding for FLAG‐PKD1/2/3 WT (pdcDNA‐FLAG‐PRKD1/2/3) and GST‐PKD1/2 (pDEST27‐PRKD2) have been described previously 36. Mutagenesis of pDONR223‐PRKD1 was carried out by site directed mutagenesis via the Quikchange kit (Agilent, Santa Clara, CA, USA) for PRKD1.C705R (FW: CACATTTTCTGGTTTGAGGTCACGGTGAACGATATTTTTAAAATGAA, RV: TTCATTTTAAAAATATCGTTCACCGTGACCTCAAACCAGAAAATGTG) and for PRKD1.Y749F (FW: CAGGAGCCAGGAAAGCGGGGGTACC, RV: GGTACCCCCGCTTTCCTGGCTCCTG). Expression clones were generated via the Gateway LR reaction. For the expression of PKD1 catalytic domain in Sf21 insect cells, PKD1 was cloned into the pI‐SUMOstar vector (Lifesensors, Malvern, PA, USA) using the following primers: FW: GAACAGATTGGAGGTATTTTTCCTGATGAAGTACTGGGT and RV: CCGCGGCCGCTCTAGTTATTATCAGAGGATGCTGACACGCTC. The ORF was then introduced in a Bacmid by transforming DH10Bac cells. Clones were selected by absence of X‐gal activity. For the expression of MBP‐tagged catalytic domain of mPKD1 in BL21 Rosetta cells mPKD1‐CAT was cloned via In‐Fusion in pMAL‐C2X using the following primers: FW:TTCAGAATTCGGATCCGTGGATATCAGCACAGTCTATCA and RV:AGTGCCAAGCTTGCCTGCAGTCAGAGGATGCTGACACGC.

### Protein expression, purification and pull‐down experiments

Transient transfections of HEK293 cells were carried out with polyethylene‐imine (PEI) at a 1:3 (m/m) plasmid/PEI ratio. Fourty‐eight hours post‐transfection, cells were lysed in 50 mm Tris, pH 7.4, 150 mm NaCl, 15 mm EDTA, 1% NP‐40 supplemented with phosphatase inhibitors (Phosphostop, Roche, Mannheim, Germany), and protease inhibitors (cOmplete, Roche). Cell lysates were incubated with affinity beads for 2 h at 4 °C while rotating [Glutathione Sepharose 4B (GE healthcare) or anti‐FLAG M2 beads (Sigma)]. Next, the beads were washed twice with NENT2000 (50 mm Tris, pH 7.4, 1 mm EDTA, 2000 mm NaCl, 0.1% NP40, 25% glycerol) unless stated otherwise, and once with NENT100 (cf. NENT2000 but containing 100 mm NaCl). Elution was done in 50 mm Tris.HCl pH7.4, 50 mm NaCl, 25% glycerol using competing peptide (150 ng·μL^−1^ 3xFLAG peptide for FLAG pulldowns) or 20 mm glutathione (GST‐pulldowns). Protein purity and concentrations were determined on an SDS/PAGE using a BSA standard.

Endogenous PKD1 was precipitated from HEK293 cells using a home‐made antibody that recognizes the C‐terminal epitope in PKD1 (EEREMKALSERVSIL). Cell lysate was incubated with antibody for 2 h and protein A beads were subsequently added. Beads were washed two times with NENT750 (50 mm Tris, pH 7.4, 1 mm EDTA, 750 mm NaCl, 0.1% NP40, 25% glycerol) and once with TBS (50 mm Tris, pH 7.4, 150 mm NaCl) prior to on‐bead kinase activity assays.

Exogenous expression and purification of 6xHis‐SUMO‐PKD1‐CAT in Sf21 insect cells was done according to standard procedures. Briefly, 6xHis‐SUMO‐PKD1‐CAT was expressed by addition of recombinant baculovirus to a Sf21 cell culture at a cell density of 1 million cells per milliliter and a multiplicity of infection of around 1. Cell culture pellets were harvested 72 h after infection by centrifugation at 10 000 ***g***. For protein purification, the pellet was resuspended in 50 mm Tris pH8, 300 mm NaCl, 10% glycerol supplemented with phosphatase inhibitors (Phosphostop, Roche), and protease inhibitors (cOmplete, Roche). Cells were subsequently lysed by 20 strokes in a Dounce homogenizer. The supernatant was collected by centrifugation at 15 000 ***g*** for 30 min and loaded on a Ni^2+^‐NTA agarose column. After flowthrough of the soluble fraction, the column was washed with 10 column volumes of 50 mm Tris pH8, 750 mm NaCl, 20 mm Imidazole, 10% glycerol and 5 column volumes of 50 mm Tris pH8, 50 mm NaCl, 20 mm Imidazole, 10% glycerol. Proteins were eluted in 50 mm Tris pH8, 50 mm NaCl, 160 mm Imidazole, 10% glycerol. Abundance and purity of proteins in the elution fractions was verified by SDS/PAGE and peak fractions were pooled and dialyzed against 50 mm Tris pH8, 50 mm NaCl, 25% glycerol.

Exogenous expression of MBP‐tagged mouse PKD1 catalytic domain in BL21‐Rosetta cells was done according to standard procedures. Briefly, BL21‐Rosetta cells were transformed with pMAL‐C2X‐mPKD1CAT and an overnight preculture was inoculated in 1L LB containing 100 μg·mL^−1^ ampicillin and 25 μg·mL^−1^ chloramphenicol. At OD600 of 0.6 expression of MBP‐mPKD1‐CAT was induced with 0.5 mm IPTG. Four hours post induction the cell pellet was collected by centrifugation at 4000 g for 20 min. MBP‐mPKD1‐CAT was purified according to the pMAL Protein Fusion and Purification System manual (NEB, Ipswich, MA, USA). Peak elution fractions were pooled and dialyzed against 50 mm Tris pH8, 50 mm NaCl, 25% glycerol.

### Activity assays and kinetics

For *in vitro* autophosphorylation of PKDs, purified protein was incubated with 100 μm ATP in 50 mm Tris, pH7.4, 10 mm MgCl_2_ and allowed to incubate for 30 min or the indicated time points at 30 °C. Reactions were stopped by addition of SDS sample buffer and boiling at 95 °C for 5 min prior to loading of an SDS/PAGE. Small unilamellar vesicles (SUVs) containing PS/PDB were added to the reaction mixture as indicated. Vesicles were prepared as follows: PDB and PS were mixed to final concentrations of 100 μg·mL^−1^ PS and 250 nM PDB and dried using a Savant Speedvac concentrator (Thermo Fisher Scientific). Dried lipids were resuspended in 50 mm Tris pH7.4 and sonicated 3 times for 10 min, vesicle fragmentation was verified by the appearance of a clear solution.

For kinase assays where Syn‐2 phosphorylation was assessed, the following reaction mixture was prepared: 50 mm Tris, pH7.4, 10 mm MgCl_2_, 50 ng PKD and 1.5 mg·mL^−1^ Syn‐2 peptide. Reactions were started with 100 μm ATP complemented with 2 μCi [γ‐^32^P]ATP (Perkin‐Elmer, Waltham, MA, USA). After 10′ (in the linear range) the reaction was stopped by spotting 30 μL on a Whatman P81 filter paper. The filter papers were washed 3 times in 0.5% phosphoric acid, followed by one wash in 100% acetone. Subsequently the papers were air‐dried and counted using the Tri‐Carb 2810 TR scintillation counter (Perkin‐Elmer). Data were analyzed using graphpad (PRISM, La Jolla, CA, USA).

### Mass spectrometric analysis of Tyr phosphorylation sites

20 μg of recombinant PKD, incubated with and without ATP for 60 min, were precipitated 37 and digested with trypsin (0.4 μg, overnight, 37 °C). The resulting peptide mixture was desalted on C18 Micro Spin Columns (Harvard Apparatus) before being subjected to anti‐pTyr IP (PY99, Santa Cruz Biotechnology, Dallas, TX, USA) in TBS/1% n‐octyl glucoside (overnight, 4 °C). Beads were eluted with 50% acetonitrile/1% formic acid (10 min, RT). Subsequently, samples were prepared for MS by using C18 ZipTips. The resulting peptide mixture was submitted to high resolution LC‐MS/MS using an Ultimate 3000 nano UPLC system interfaced with an Orbitrap Q‐Exactive MS via an EASY‐spray (C18, 15 cm) column (Thermo Fisher Scientific). The Q‐Exactive MS was operated in data‐dependent mode selecting the top ten precursors for MS/MS. Protein identifications were obtained from the mascot (Matrix science, version 2.2.2) search engine using UniProt/SwissProt (*Homo sapiens*, 20202 entries) as a database, allowing up to three missed tryptic cleavages, phosphorylation of STY and oxidation of Met as variable modifications. Peptide abundances were determined using the progenesis qi software package (Nonlinear Dynamics, Newcastle upon Tyne, UK).

## Results

### PKD autophosphorylates on Tyr in the P + 1 loop *in vitro*

**Figure 1:**
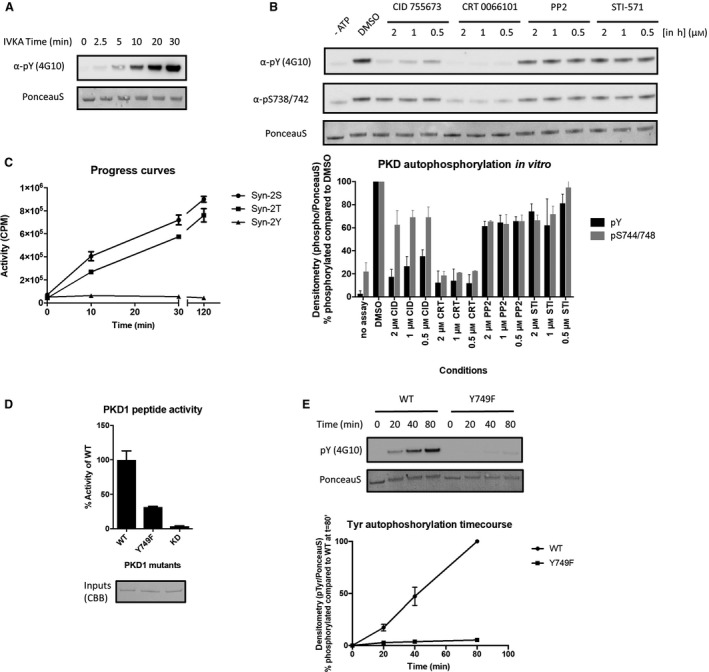
Protein kinase D displays autophosphorylation on Tyr residues. (A) Time course of PKD1 phosphorylation on Tyr residues by PKD1 *in vitro*. FLAG‐PKD1 was purified from HEK293 cells as described in the Materials and methods section, incubated with Mg2+.ATP for the indicated timepoints and assayed for Tyr phosphorylation via immunoblotting. (B) *in vitro* kinase assay (IVKA) for PKD1 autophosphorylation in presence of the indicated inhibitors. Quantification of two individual experiments is shown. Graphs represent mean ± SEM. (C) Progress curves of Syntide‐2 phosphorylation by PKD1. FLAG‐PKD1 purified from HEK293 cells was incubated with one of three different Syntide‐2 derived peptides; where Syn‐2S is a peptide containing Ser as phospho‐acceptor, Syn‐2T contains a Thr and Syn‐2Y contains a Tyr as phospho‐acceptor. (D) Activity of Y749F and kinase‐dead K612A mutants towards Syntide‐2. FLAG‐tagged PKD1 WT and K612A or Y749F mutants were incubated with Syn‐2 peptide and activity was measured in a radiometric kinase assay. (E) Autophosphorylation of PKD1 Y749F on Tyr residues. FLAG‐PKD1 WT and a Y749F mutant were incubated with Mg2+.ATP for the indicated time points and assayed for Tyr phosphorylation via immunoblotting. Quantification of three individual experiments is shown. Graphs represent mean SEM.

When studying Tyr phosphorylation of PKD by tyrosine kinases *in vitro*, we observed that when PKD was incubated with Mg^2+^.ATP alone, high levels of Tyr phosphorylation were detected without addition of an upstream kinase. As this phenomenon could be due to the co‐precipitation of a Tyr kinase during PKD purification, we purified PKD using high ionic‐strength washing steps (2M NaCl) obtaining a highly pure PKD preparation (100% on Coomassie Brilliant Blue stained SDS/PAGE, Fig. S1), devoid of any tyrosine kinases as determined via Mass‐spectrometry (Table S1). Yet, this PKD preparation was still phosphorylated on Tyr residues upon incubation with Mg^2+^.ATP (Fig. 1A). Therefore, we hypothesized that PKD might autophosphorylate on Tyr residues in *vitro*.

To test this hypothesis, we followed *in vitro* Tyr phosphorylation of PKD1 in presence of the PKD inhibitors CRT0066101 and CID755673. Interestingly, we observed a dose‐dependent decrease of Tyr phosphorylation upon incubation with these PKD inhibitors, while the same Tyr phosphorylation *in vitro* was insensitive to inhibition by the tyrosine kinase inhibitors PP2 and STI‐751 (Fig. 1B). The observed phenomenon was not an artefact of PKD overexpression in human cells, since both endogenously expressed PKD1 retrieved from cells as well as heterologously expressed PKD1 catalytic domain in *Spodoptera frugiperda* Sf21 insect cells displayed autophosphorylation activity towards Tyr residues (Fig. S2). Furthermore, all three PKD isoforms displayed *in vitro* autophosphorylation on Tyr residues, albeit more pronounced for PKD2 (Fig. S3). PKD Tyr kinase activity was restricted to autophosphorylation, since we could not detect any activity towards a modified Syntide‐2 peptide containing a Tyr in lieu of a Ser as the phospho‐acceptor (Fig. 1C).

Next, we wondered which Tyr sites were autophosphorylated by PKD during the *in vitro* reaction. To identify these sites, we performed an MS analysis of full‐length PKD1 subjected to a terminal autophosphorylation reaction. Interestingly, only one Tyr phosphorylation site was identified, namely Tyr‐749 in the P + 1 loop of the kinase (Table 1). This was confirmed using a mutant in which this residue was substituted with Phe (PKD1 Y749F mutant). Although the activity of a P + 1 loop Y‐F mutant is strongly impaired, it still displays some trans‐phosphorylation activity (Fig. 1D and 36). However the mutant failed to autophosphorylate on Tyr residues even after 80 min in the reaction (Fig. 1E).

**Table 1:** Mass‐spectrometric analysis of Tyr‐phosphorylated peptides in the PKD autophosphorylation reaction. Peptide abundances were determined via the progenesis qi software package

Sequence	Peptide mascot score (highest abundance)	Modifications	Raw peptide abundance	
			no RXN	60′ auto‐P
SVVGTPAYLAPEVLR	82.54	[8] Phospho (Y)	982.5287	15084355
SVVGTPAYLAPEVLR	50.08	[8] Phospho (Y)	844.3315	1280638
RSVVGTPAYLAPEVLR	49.66	[9] Phospho (Y)	175.3688	503364.6
RSVVGTPAYLAPEVLR	44.1	[6] Phospho (ST)|[9] Phospho (Y)	6470.141	317003

### Molecular determinants of Tyr autophosphorylation

**Figure 2:**
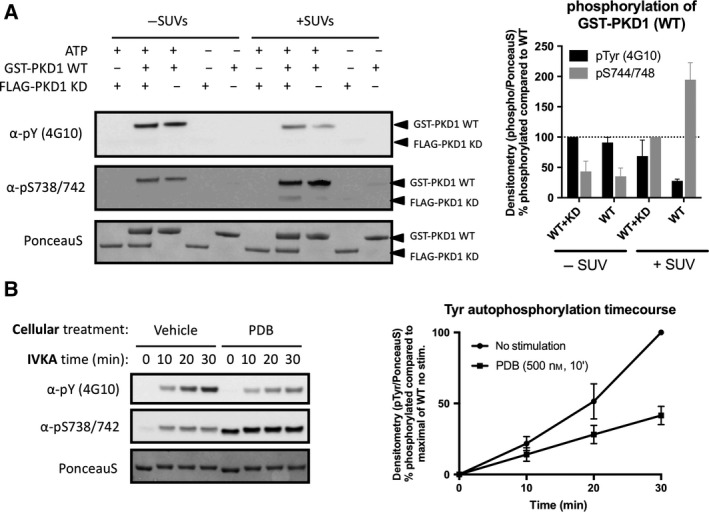
Structural determinants for Tyr autophosphorylation. (A) PKD autophosphorylates on Tyr in *cis* and Tyr kinase activity is inhibited in presence of PS/PDB. GST‐tagged PKD WT and FLAG‐tagged PKD KD were incubated separately or together in presence or absence of ATP and allowed to react for 30′ at 30 °C. Quantification of three individual experiments is shown. Graphs represent mean ± SEM. (B) Time course of *in vitro* autophosphorylation by FLAG‐PKD1 purified from unstimulated or cells stimulated with PDB (500 nm, 10′). PKD1 was incubated with Mg2+.ATP for the indicated time points and assayed for Tyr and Ser‐738/742 phosphorylation via immunoblotting. Quantification of three individual experiments is shown. Graphs represent mean ± SEM.

Next, we wondered under which conditions PKDs are able to autophosphorylate on Tyr residue(s). Autophosphorylation can occur in *cis* or in *trans*. To determine which mechanism applies to PKD Tyr‐autophosphorylation, we incubated GST‐tagged wild‐type PKD1 with a FLAG‐tagged kinase‐dead (KD) PKD1 mutant, in order to be able to distinguish them by molecular weight. As shown in Fig. 2A, GST‐PKD autophosphorylated efficiently on Tyr residues, but no Tyr phosphorylation could be detected for the FLAG‐tagged KD construct, indicating that the autophosphorylation reaction on Tyr occurs in *cis*. Autophosphorylation of the activation loop Ser‐738/742 residues also occurred predominantly in *cis* in this reaction (Fig. 2A). Stimulation with small unilamellar vesicles (SUVs) consisting of phosphatidylserine/Phorbol‐12,13‐dibutyrate (PS/PDB) resulted in an expected increase in activation loop Ser‐738/742 autophosphorylation (Fig. 2A), as previously described 29. Surprisingly however, Tyr autophosphorylation activity was reduced after lipid stimulation (Fig. 2A), indicating that lipid‐induced conformational changes might structure PKD towards Ser autophosphorylation, while a ‘lipid‐devoid’ conformation would promote Tyr autophosphorylation. This was further confirmed by following the kinetics of PKD purified from PDB‐stimulated cells, indicating that indeed, the lipid‐activated conformation of PKD disfavors Tyr autophosphorylation (Fig. 2B).

### Cysteine in the catalytic loop is a major determinant for Tyr autophosphorylation activity

**Figure 3:**
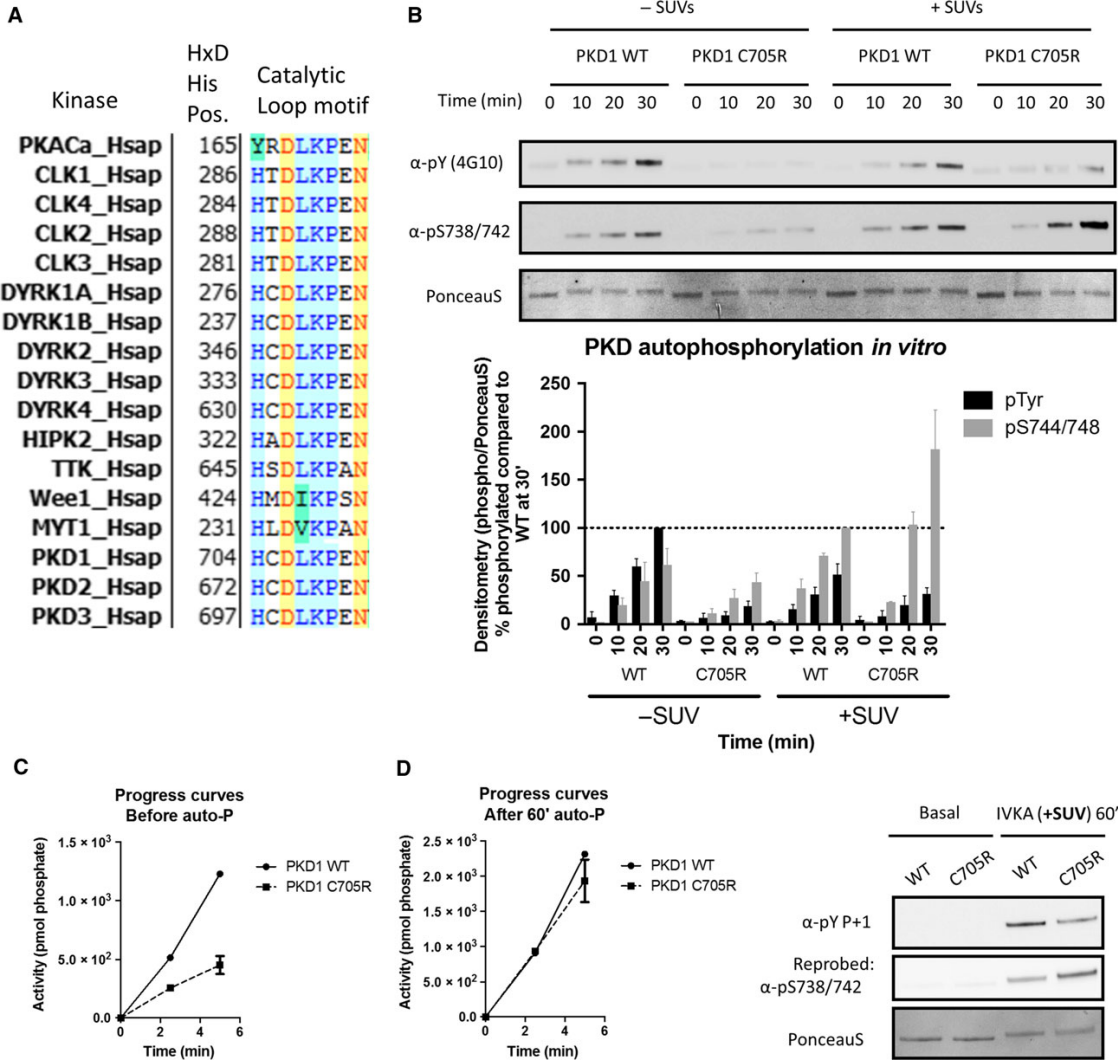
A Protein kinase D C705R mutant results in altered enzymatic properties. (A) Multiple sequence alignment of several reported dual‐specificity kinases that have substitutions for the conserved Arg in the HRD motif. Alignment was performed in Vector NTi using the alignX module. (B) Ser and Tyr autophosphorylation activities of PKD WT and the C705R mutant in presence or absence of PS/PDB containing SUVs. FLAG‐PKD1 WT and a C705R mutant were purified from HEK293 cells as described in the Materials and methods section, incubated with Mg2+.ATP for the indicated time points and assayed for Tyr and Ser‐738/742 phosphorylation via immunoblotting. Quantification of three individual experiments is shown. Graphs represent mean ± SEM. (C) Activities toward Syn‐2 of PKD WT and C705R in the inactive state of PKD. (D) Activities toward Syn‐2 of PKD WT and C705R after 60 min of prior autophosphorylation in presence of PS/PDB containing SUVs. A blot representing the phosphorylation state of PKD1 preparations used for C and D is shown in the right panel of Fig. 3D.

The intriguing property of Tyr autophosphorylation, which is only reported for a small subset of Ser/Thr kinases, raised the question which structural features in PKD might contribute to its Tyr kinase activity. Interestingly, in 1992, Tony Hunter already predicted linear motifs that could contribute to dual specificity of protein kinases 38. One motif in which he observed divergence from ‘normal’ Ser/Thr kinases was the catalytic loop, where many dual‐specificity kinases have substitutions for the conserved Arg in the HRD motif. Intriguingly, alignment of currently reported Ser/Thr kinases that exhibit dual specificity indeed confirmed that many of them have substitutions for the conserved Arg residue (Fig. 3A). This includes PKDs, which contain a Cys residue at this Arg position. Mutation of Cys to Arg in PKD1 (PKD1.C705R) resulted in an interesting enzymatic profile. In resting conditions, the activity of this mutant was decreased compared to WT PKD1: it showed less Tyr and Ser autophosphorylation compared to WT (Fig. 3B), but also the rate of Syn‐2 phosphorylation was decreased compared to WT in the resting state (Fig. 3C). Upon stimulation with PS/PDB containing vesicles, activation loop Ser autophosphorylation activity potently increased in the C705R mutant, reaching even higher levels than the WT kinase, while still showing low levels of Tyr autophosphorylation (Fig. 3B). Activities towards Syn‐2 were equal for both the WT and mutant after prior *in vitro* autophosphorylation, despite the higher activation loop Ser phosphorylation in the mutant (Fig. 3D). These data indicate that the residue at the HRD Arg position may not only stabilize the inactive conformation, but also the direct the specificity of the autophosphorylation reaction.

### Tyr autophosphorylation in cells is inhibited by a factor associating with PKD

**Figure 4:**
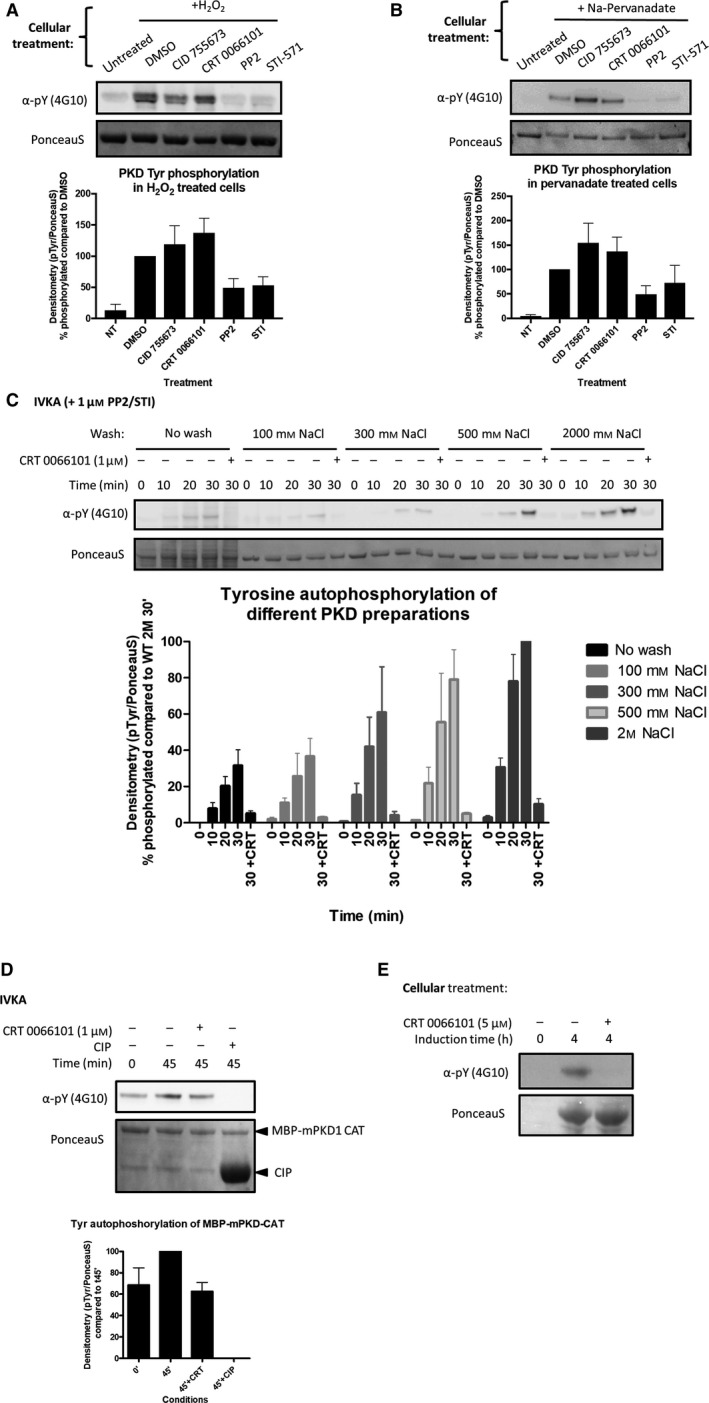
Protein kinase D associates with a factor in cells that impedes its autophosphorylation at Tyr residues. (A) Tyr phosphorylation of PKD after stimulation of HEK293 cells with H_2_O_2_ (10 mm, 10 min) in the presence of the indicated kinase inhibitors (5 μm). FLAG‐PKD1 was precipitated from cells and the Tyr phosphorylation state was assessed by immunoblotting. Quantification of three individual experiments is shown. Graphs represent mean ± SEM. (B) Tyr phosphorylation of PKD after stimulation with pervanadate (75 μm, 30 min) in the presence of the indicated kinase inhibitors (5 μm). FLAG‐PKD1 was precipitated from HEK293 cells and the Tyr phosphorylation state was assessed by immunoblotting. Quantification of three individual experiments is shown. Graphs represent mean ± SEM. (C) Autophosphorylation on Tyr residues of differentially purified PKD preparations. For details see text. Quantification of three individual experiments is shown. Graphs represent mean ± SEM. (D) Tyr autophosphorylation of MBP‐mPKD1‐CAT purified from bacteria. MBP‐mPKD1‐CAT was incubated with Mg2+.ATP for 45 min in presence or absence of PKD inhibitor or Calf Intestine Alkaline Phosphatase (CIP) and assayed for Tyr phosphorylation via immunoblotting. Quantification of three individual experiments is shown. Graphs represent mean ± SEM. (E) Expression of MBP‐mPKD1‐CAT in BL21‐Rosetta cells in presence or absence of the PKD inhibitor CRT 0066101. BL21 cells harboring pMALC2X‐MBP‐mPKD1‐CAT were grown and at OD600 of 0.6 the culture was induced with 0.5 mm IPTG and divided to either incubate with or without 5 μm CRT 0066101. Four hours post induction cell pellets were collected and analyzed for Tyr phosphorylation by immunoblotting.

Since PKDs display this unusual activity *in vitro*, we wondered whether autophosphorylation was also occurring in intact cells under conditions where PKDs are known to be tyrosine phosphorylated. Therefore, we treated cells with the Tyr kinase activator/Tyr phosphatase inhibitor H_2_O_2_ (Fig. 4A) or the Tyr phosphatase inhibitor pervanadate (Fig. 4B) and probed for the sensitivity of PKD1 Tyr phosphorylation to the same set of inhibitors as in Fig. 1C. Under these conditions, it was clear that Tyr phosphorylation of PKD1 was mediated exclusively by upstream tyrosine kinases, which were responsive to PP2 and STI‐571 inhibition, and not via autophosphorylation, since Tyr phosphorylation was insensitive to PKD inhibition.

We then wondered why this intracellular PKD activity was so divergent from its *in vitro* activity. Since it was clear that a lack of Tyr autophosphorylation in cells is not due to high phosphatase activity, we hypothesized that an inhibitory factor associating with PKD might block its autocatalytic activity in cells. In order to test this hypothesis, we produced different PKD1 preparations, each subjected to washing steps of different ionic strengths. If a cofactor dependent on ionic interactions bound to PKD, it could progressively dissociate with increasing strength of the washing buffer, which would subsequently increase PKD Tyr autophosphorylation. Tyr autophosphorylation by these differentially purified PKD preparations was followed in the presence of PP2 and STI‐571 to exclude any phosphorylation exerted by a co‐purifying Tyr kinase. The PKD inhibitor CRT0066101 was included as a control to verify that the observed Tyr phosphorylation was indeed dependent on PKD activity. As shown in Fig. 4C, Tyr autophosphorylation increased with increased ionic strength in washing steps, indicative of an inhibitory factor associating with PKD in cells via ionic interactions.

Additionally, when we purified the catalytic domain of PKD1 from bacterial cells to assess *in vitro* Tyr autophosphorylation activity, we found that the protein was recovered as already being Tyr phosphorylated, while still showing a noticeable (albeit modest) increase in Tyr autophosphorylation *in vitro* (Fig. 4D). Since bacteria contain only a few ‘BY’ Tyr kinases (that do not share the conserved kinase fold) we wondered whether bacterial Tyr phosphorylation of PKD1 was the result of autophosphorylation activity. Therefore we induced MBP‐PKD1‐CAT expression in presence or absence of the PKD inhibitor CRT0066101. PKD inhibition during induction resulted in a complete loss of Tyr phosphorylation, indicating that PKD1 can autophosphorylate on Tyr residues in bacterial cells (Fig. 4E). These results are supportive of either the absence of an auto‐inhibitory factor or decreased Tyr phosphatase activity in bacteria; although it should be noted that loss of phosphatase activity by itself does not increase Tyr autophosphorylation in eukaryotic cells (Fig 4A and 4B).

## Discussion

Protein kinases often critically depend on phosphorylation for their regulation. In a cellular context, these regulatory phosphorylations are frequently exerted by upstream kinases. Many kinases however also possess the ability to autophosphorylate, and a recent study suggests that this might be more frequent than hitherto believed 4. Protein kinase D is also regulated via phosphorylation and can autophosphorylate both *in vitro* and in cells. Known *in vitro* autophosphorylation sites for PKD include the activation loop Ser‐738/742 residues and the C‐terminal Ser‐910 residue 29, 31, 39, 40. In cells, PKD is phosphorylated on Ser‐738 mainly by upstream PKCs, while Ser‐742 phosphorylation can occur as a PKC‐mediated event, but under certain circumstances also as an autocatalytic event 41.

**Figure 5:**
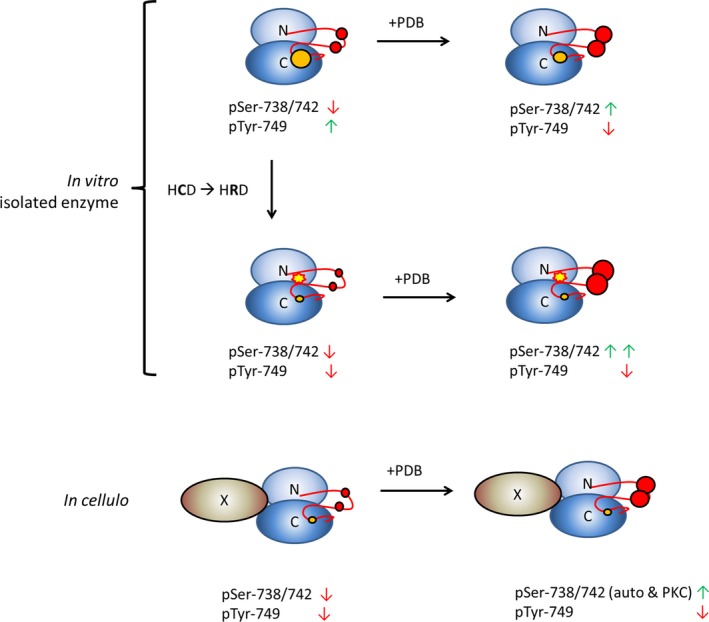
Mechanisms that regulate PKD Tyr autophosphorylation. Modalities in which PKD can acquire Tyr specificity. PKD purified from resting cells displays potent Tyr autophosphorylation. Ser‐738/742 autophosphorylation is still at low levels in an unstimulated enzyme. Activation with PDB results in increased Ser‐738/742 autophosphorylation and decreased Tyr autophosphorylation activity. Mutation of Cys to Arg in the catalytic loop HCD motif results in loss of Tyr and Ser‐738/742 autophosphorylation in unstimulated conditions and an increase in PDB stimulated Ser‐738/742 autophosphorylation. Cellular autophosphorylation activity is likely suppressed by an inhibitory factor (X) associating with PKD.

In this study, we describe the surprising finding that PKDs can also autophosphorylate at a Tyr residue in the P + 1 loop. This residue lies at an interesting position, just before the APE motif and is highly conserved in many Ser/Thr kinases. In a previous study, we found that transphosphorylation of this residue by Abl results in higher turnover of substrate phosphorylation 36. Interestingly, the enzymatic requirements for this Tyr autophosphorylation significantly differ from those for activation loop Ser autophosphorylation. Indeed, we observed that while PKD Ser autophosphorylation is stimulated in the presence of PS/PDB containing SUVs, or when previously activated by PDB in cellulo, Tyr autophosphorylation activity is inhibited in these conditions (Fig. 2A and schematic representation in Fig. 5). Furthermore, it seems that Tyr autophosphorylation displays a high sensitivity to CID755673‐mediated inhibition, while activation loop Ser autophosphorylation is largely unaffected by this PKD inhibitor (Fig. 1C). This corroborates cellular data showing that, with this allosteric compound, activation loop phosphorylation in cells is not diminished but rather increased 42.

Interestingly, in a cellular context, the phosphorylation at Tyr‐749 does not seem to be catalyzed in auto, but by bona‐fide upstream Tyr kinases. In oxidative stress conditions, for example, we could show that Abl is an upstream kinase for the P + 1 Tyr residue, and that it specifically phosphorylates the PKD2 isoform due to an isoform‐specific motif preceding the Tyr residue 36. The mechanism that prohibits Tyr autophosphorylation in cells is hitherto unclear, but it likely involves the association of an inhibitory factor (Fig. 5). It is indeed not uncommon that (Tyr) autophosphorylation reactions are regulated by binding of cofactors. For example, GSK3β can autophosphorylate on Tyr residues, an activity that is dependent on Hsp90 association 18. Furthermore, p38α autophosphorylation in cells is dependent on association with tumor growth factor‐β‐activated kinase 1/MAP3K7‐binding protein 1 (TAB 1)^43^. In PKD, autophosphorylation on Ser‐742 in cells seems to be dependent on the association with certain Gα isoforms 44.

In search for a structural explanation for its intrinsic dual‐specificity, we found that PKD does not contain a conserved Arg residue in the catalytic loop HRD motif; instead, it contains a Cys. Interestingly, HRD Arg substitutions are shared by many kinases with reported dual specificity (Fig. 3A). Substitution of Cys with Arg in PKD resulted in an overall loss of activity in the auto‐inhibited state, a loss of Tyr autophosphorylation in both lipid‐stimulated as well as unstimulated conditions and increased Ser autophosphorylation in PDB stimulated conditions (Figs 3B and 5). The Arg occurring in most kinases is adjacent to the catalytic Asp and stabilizes the activation loop in the active state by forming a basic pocket (together with a Lys residue C‐terminal of the DFG motif) to bind the primary phospho‐acceptor in the activation loop 45. PKD is a non‐RD kinase, but is nonetheless dependent on activation loop phosphorylation for its activity 34. Interestingly, the Arg residue in the HRD motif is sometimes seen to pair with a conserved Glu in the αC helix in inactive kinases 2, 46. This highly conserved Glu forms the characteristic salt bridge with Lys in β strand 3 of the N‐lobe in active kinases. A substitution of Cys with Arg in PKD could potentially result in the formation of such an inhibitory Arg‐Glu salt bridge and stabilize the inactive conformation.

In conclusion, we report an unusual Tyr autophosphorylation activity by the PKD family of Ser/Thr kinases, an activity that has catalytic requirements distinct from its Ser autophosphorylation activities, and is dependent on the presence of a Cys residue in the HxD catalytic loop motif. Since this activity is only observed *in vitro* and suppressed in a cellular context, future research efforts will need to be designated to determine under which conditions Tyr autophosphorylation may be allowed to occur in a cellular context.

## Author contributions

MC designed and executed experiments and wrote the manuscript. RD and EW were responsible for Mass‐Spec experiments and subsequent data analysis. VJ, PJP, and JVL supervised the project and revised the manuscript.

## Supporting information


**Fig. S1.** Purity of PKD preparations assessed on a Coomassie brilliant blue stained polyacrylamide gel.
**Fig. S2.** Tyr autophosphorylation activity of endogenous PKD precipitated from HEK293 cells (upper panel) and PKD kinase domain expressed in insect cells (lower panel).
**Fig. S3.** Tyr autophosphorylation activity of PKD isoforms *in vitro*.
**Table S1.** List of proteins identified in a highly pure PKD preparation (washed with a buffer containing 2M NaCl) and their mascot scores.

## References

[1] Gross S , Rahal R , Stransky N , Lengauer C and Hoeflich KP (2015) Targeting cancer with kinase inhibitors. J Clin Investig 125, 1780–1789. 25932675 10.1172/JCI76094 PMC4463189

[2] Nolen B , Taylor S and Ghosh G (2004) Regulation of protein kinases; controlling activity through activation segment conformation. Mol Cell 15, 661–675. 15350212 10.1016/j.molcel.2004.08.024

[3] Johnson LN and Lewis RJ (2001) Structural basis for control by phosphorylation. Chem Rev 101, 2209–2242. 11749371 10.1021/cr000225s

[4] Beenstock J , Mooshayef N and Engelberg D (2016) How do protein kinases take a selfie (Autophosphorylate)? Trends Biochem Sci 41, 938–953. 27594179 10.1016/j.tibs.2016.08.006

[5] Lemmon MA and Schlessinger J (2010) Cell signaling by receptor tyrosine kinases. Cell 141, 1117–1134. 20602996 10.1016/j.cell.2010.06.011 PMC2914105

[6] Manning G , Whyte DB , Martinez R , Hunter T and Sudarsanam S (2002) The protein kinase complement of the human genome. Science 298, 1912–1934. 12471243 10.1126/science.1075762

[7] Zhu H , Klemic JF , Chang S , Bertone P , Casamayor A , Klemic KG , Smith D , Gerstein M , Reed MA and Snyder M (2000) Analysis of yeast protein kinases using protein chips. Nat Genet 26, 283–289. 11062466 10.1038/81576

[8] Vilk G , Weber JE , Turowec JP , Duncan JS , Wu C , Derksen DR , Zien P , Sarno S , Donella‐Deana A , Lajoie G *et al* (2008) Protein kinase CK2 catalyzes tyrosine phosphorylation in mammalian cells. Cell Signal 20, 1942–1951. 18662771 10.1016/j.cellsig.2008.07.002

[9] Lundgren K , Walworth N , Booher R , Dembski M , Kirschner M and Beach D (1991) mik1 and wee1 cooperate in the inhibitory tyrosine phosphorylation of cdc2. Cell 64, 1111–1122. 1706223 10.1016/0092-8674(91)90266-2

[10] Mueller PR , Coleman TR , Kumagai A and Dunphy WG (1995) Myt1: a membrane‐associated inhibitory kinase that phosphorylates Cdc2 on both threonine‐14 and tyrosine‐15. Science 270, 86–90. 7569953 10.1126/science.270.5233.86

[11] West AB , Moore DJ , Biskup S , Bugayenko A , Smith WW , Ross CA , Dawson VL and Dawson TM (2005) Parkinson's disease‐associated mutations in leucine‐rich repeat kinase 2 augment kinase activity. Proc Natl Acad Sci U S A. 102, 16842–16847. 16269541 10.1073/pnas.0507360102 PMC1283829

[12] Lawler S , Feng XH , Chen RH , Maruoka EM , Turck CW , Griswold‐Prenner I and Derynck R (1997) The type II transforming growth factor‐beta receptor autophosphorylates not only on serine and threonine but also on tyrosine residues. J Biol Chem 272, 14850–14859. 9169454 10.1074/jbc.272.23.14850

[13] Wang QM , Fiol CJ , DePaoli‐Roach AA and Roach PJ (1994) Glycogen synthase kinase‐3 beta is a dual specificity kinase differentially regulated by tyrosine and serine/threonine phosphorylation. J Biol Chem 269, 14566–14574. 7514173

[14] Siepi F , Gatti V , Camerini S , Crescenzi M and Soddu S (2013) HIPK2 catalytic activity and subcellular localization are regulated by activation‐loop Y354 autophosphorylation. Biochim Biophys Acta 1833, 1443–1453. 23485397 10.1016/j.bbamcr.2013.02.018 PMC3787740

[15] Toshima J , Tanaka T and Mizuno K (1999) Dual specificity protein kinase activity of testis‐specific protein kinase 1 and its regulation by autophosphorylation of serine‐215 within the activation loop. J Biol Chem 274, 12171–12176. 10207045 10.1074/jbc.274.17.12171

[16] Bullock AN , Das S , Debreczeni JE , Rellos P , Fedorov O , Niesen FH , Guo K , Papagrigoriou E , Amos AL , Cho S *et al* (2009) Kinase domain insertions define distinct roles of CLK kinases in SR protein phosphorylation. Structure. 17, 352–362. 19278650 10.1016/j.str.2008.12.023 PMC2667211

[17] Liu X and Winey M (2012) The MPS1 family of protein kinases. Annu Rev Biochem 81, 561–585. 22482908 10.1146/annurev-biochem-061611-090435 PMC4026297

[18] Lochhead PA , Kinstrie R , Sibbet G , Rawjee T , Morrice N and Cleghon V (2006) A chaperone‐dependent GSK3beta transitional intermediate mediates activation‐loop autophosphorylation. Mol Cell 24, 627–633. 17188038 10.1016/j.molcel.2006.10.009

[19] Lochhead PA , Sibbet G , Morrice N and Cleghon V (2005) Activation‐loop autophosphorylation is mediated by a novel transitional intermediate form of DYRKs. Cell 121, 925–936. 15960979 10.1016/j.cell.2005.03.034

[20] Plaza‐Menacho I , Barnouin K , Barry R , Borg A , Orme M , Chauhan R , Mouilleron S , Martínez‐Torres RJ , Meier P and McDonald NQ (2016) RET functions as a dual‐specificity kinase that requires allosteric inputs from juxtamembrane elements. Cell Rep 17, 3319–3332. 28009299 10.1016/j.celrep.2016.11.061 PMC5199340

[21] Yokoyama N , Lougheed J and Miller WT (2005) Phosphorylation of WASP by the Cdc42‐associated kinase ACK1: dual hydroxyamino acid specificity in a tyrosine kinase. J Biol Chem 280, 42219–42226. 16257963 10.1074/jbc.M506996200

[22] Rozengurt E , Rey O and Waldron RT (2005) Protein kinase D signaling. J Biol Chem 280, 13205–13208. 15701647 10.1074/jbc.R500002200

[23] Fu Y and Rubin CS (2011) Protein kinase D: coupling extracellular stimuli to the regulation of cell physiology. EMBO Rep 12, 785–796. 21738220 10.1038/embor.2011.139 PMC3147268

[24] Iglesias T and Rozengurt E (1999) Protein kinase D activation by deletion of its cysteine‐rich motifs. FEBS Lett 454, 53–56. 10413094 10.1016/s0014-5793(99)00772-3

[25] Van Lint JV , Sinnett‐Smith J and Rozengurt E (1995) Expression and characterization of PKD, a phorbol ester and diacylglycerol‐stimulated serine protein kinase. J Biol Chem 270, 1455–1461. 7836415 10.1074/jbc.270.3.1455

[26] Waldron RT and Rozengurt E (2003) Protein kinase C phosphorylates protein kinase D activation loop Ser744 and Ser748 and releases autoinhibition by the pleckstrin homology domain. J Biol Chem 278, 154–163. 12407104 10.1074/jbc.M208075200

[27] Aicart‐Ramos C , He SD , Land M and Rubin CS (2016) A novel conserved domain mediates dimerization of protein kinase D (PKD) isoforms: dimerization is essential for PKD‐dependent regulation of secretion and innate immunity. J Biol Chem 291, 23516–23531. 27662904 10.1074/jbc.M116.735399 PMC5095407

[28] Steinberg SF (2012) Regulation of protein kinase D1 activity. Mol Pharmacol 81, 284–291. 22188925 10.1124/mol.111.075986 PMC3286295

[29] Rybin VO , Guo J , Harleton E , Zhang F and Steinberg SF (2012) Regulatory domain determinants that control PKD1 activity. J Biol Chem 287, 22609–22615. 22582392 10.1074/jbc.M112.379719 PMC3391082

[30] Jacamo R , Sinnett‐Smith J , Rey O , Waldron RT and Rozengurt E (2008) Sequential protein kinase C (PKC)‐dependent and PKC‐independent protein kinase D catalytic activation via Gq‐coupled receptors: differential regulation of activation loop Ser(744) and Ser(748) phosphorylation. J Biol Chem 283, 12877–12887. 18337243 10.1074/jbc.M800442200 PMC2442337

[31] Rybin VO , Guo J and Steinberg SF (2009) Protein kinase D1 autophosphorylation via distinct mechanisms at Ser744/Ser748 and Ser916. J Biol Chem 284, 2332–2343. 19029298 10.1074/jbc.M806381200 PMC2629118

[32] Storz P , Doppler H , Johannes FJ and Toker A (2003) Tyrosine phosphorylation of protein kinase D in the pleckstrin homology domain leads to activation. J Biol Chem 278, 17969–17976. 12637538 10.1074/jbc.M213224200

[33] Doppler H and Storz P (2007) A novel tyrosine phosphorylation site in protein kinase D contributes to oxidative stress‐mediated activation. J Biol Chem 282, 31873–31881. 17804414 10.1074/jbc.M703584200

[34] Storz P , Doppler H and Toker A (2004) Activation loop phosphorylation controls protein kinase D‐dependent activation of nuclear factor kappaB. Mol Pharmacol 66, 870–879. 15226414 10.1124/mol.104.000687

[35] Waldron RT and Rozengurt E (2000) Oxidative stress induces protein kinase D activation in intact cells. Involvement of Src and dependence on protein kinase C, J Biol Chem. 275, 17114–17121. 10748111 10.1074/jbc.M908959199

[36] Cobbaut M , Derua R , Döppler H , Lou HJ , Vandoninck S , Storz P , Turk BE , Seufferlein T , Waelkens E , Janssens V *et al* (2017) Differential regulation of PKD isoforms in oxidative stress conditions through phosphorylation of a conserved Tyr in the P + 1 loop. Sci Rep 7, 887. 28428613 10.1038/s41598-017-00800-w PMC5430542

[37] Wessel D and Flugge UI (1984) A method for the quantitative recovery of protein in dilute solution in the presence of detergents and lipids. Anal Biochem 138, 141–143. 6731838 10.1016/0003-2697(84)90782-6

[38] Lindberg RA , Quinn AM and Hunter T (1992) Dual‐specificity protein kinases: will any hydroxyl do? Trends Biochem Sci 17, 114–119. 1412695 10.1016/0968-0004(92)90248-8

[39] Sanchez‐Ruiloba L , Cabrera‐Poch N , Rodriguez‐Martinez M , Lopez‐Menendez C , Jean‐Mairet RM , Higuero AM and Iglesias T (2006) Protein kinase D intracellular localization and activity control kinase D‐interacting substrate of 220‐kDa traffic through a postsynaptic density‐95/discs large/zonula occludens‐1‐binding motif. J Biol Chem 281, 18888–18900. 16651260 10.1074/jbc.M603044200

[40] Kunkel MT , Garcia EL , Kajimoto T , Hall RA and Newton AC (2009) The protein scaffold NHERF‐1 controls the amplitude and duration of localized protein kinase D activity. J Biol Chem 284, 24653–24661. 19581308 10.1074/jbc.M109.024547 PMC2782054

[41] Matthews SA , Rozengurt E and Cantrell D (1999) Characterization of serine 916 as an in vivo autophosphorylation site for protein kinase D/Protein kinase Cmu. J Biol Chem 274, 26543–26549. 10473617 10.1074/jbc.274.37.26543

[42] Kunkel MT and Newton AC (2015) Protein kinase d inhibitors uncouple phosphorylation from activity by promoting agonist‐dependent activation loop phosphorylation. Chem Biol 22, 98–106. 25556943 10.1016/j.chembiol.2014.11.014 PMC4311772

[43] De Nicola GF , Martin ED , Chaikuad A , Bassi R , Clark J , Martino L , Verma S , Sicard P , Tata R , Atkinson RA *et al* (2013) Mechanism and consequence of the autoactivation of p38alpha mitogen‐activated protein kinase promoted by TAB 1. Nat Struct Mol Biol 20, 1182–1190. 24037507 10.1038/nsmb.2668 PMC3822283

[44] Waldron RT , Innamorati G , Torres‐Marquez ME , Sinnett‐Smith J and Rozengurt E (2012) Differential PKC‐dependent and ‐independent PKD activation by G protein alpha subunits of the Gq family: selective stimulation of PKD Ser(7)(4)(8) autophosphorylation by Galphaq. Cell Signal 24, 914–921. 22227248 10.1016/j.cellsig.2011.12.014 PMC3286641

[45] Steichen JM , Kuchinskas M , Keshwani MM , Yang J , Adams JA and Taylor SS (2012) Structural basis for the regulation of protein kinase A by activation loop phosphorylation. J Biol Chem 287, 14672–14680. 22334660 10.1074/jbc.M111.335091 PMC3340281

[46] Sicheri F , Moarefi I and Kuriyan J (1997) Crystal structure of the Src family tyrosine kinase Hck. Nature 385, 602–609. 9024658 10.1038/385602a0

