# Supplementary material for: Protein kinase D displays intrinsic Tyr autophosphorylation activity: insights into mechanism and regulation
Source: FEBS Lett. 2018 Jul 23;592(14):2432–43. doi: 10.1002/1873-3468.13171 (PMC6099456; doi:10.1002/1873-3468.13171)
Supplement: Supplementary file 1 — Fig. S1. Purity of PKD preparations assessed on a Coomassie brilliant blue stained polyacrylamide gel. Fig. S2. Tyr autophosphorylation activity of endogenous PKD precipitated from HEK293 cells (upper panel) and PKD kinase domain expressed in insect cells (lower panel). Fig. S3. Tyr autophosphorylation activity of PKD isoforms in vitro. Table S1. List of proteins identified in a highly pure PKD preparation (washed with a buffer containing 2M NaCl) and their mascot scores. [file FEB2-592-2432-s001.docx]

# Supplemental data


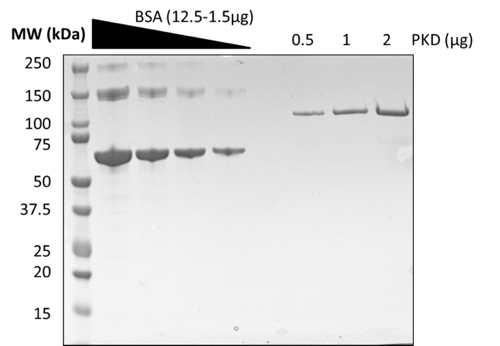


Supplemental fig. 1: purity of PKD preparations assessed on a Coomassie brilliant blue stained polyacrylamide gel.


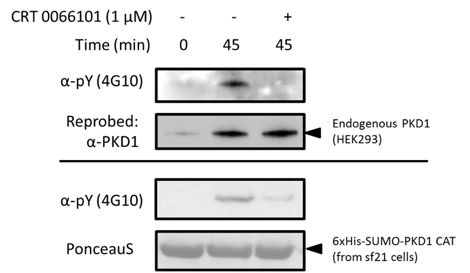


Supplemental fig. 2: Tyr autophosphorylation activity of endogenous PKD precipitated from HEK293 cells (upper panel) and PKD kinase domain expressed in insect cells (lower panel).


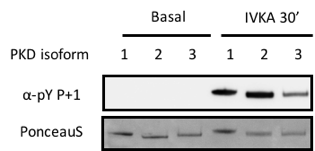


Supplemental fig. 3: Tyr autophosphorylation activity of PKD isoforms *in vitro*

Supplemental Table 1.

List of proteins identified in a highly pure PKD preparation (washed with a buffer containing 2M NaCl) and their mascot scores

| prot_hit_num | prot_acc | prot_desc | Mascot prot_score |
| --- | --- | --- | --- |
| 1 | Q15139\|KPCD1_HUMAN | Serine/threonine-protein kinase D1 OS=Homo sapiens GN=PRKD1 PE=1 SV=2 | 14149 |
| 2 | Q9BZL6\|KPCD2_HUMAN | Serine/threonine-protein kinase D2 OS=Homo sapiens GN=PRKD2 PE=1 SV=2 | 3779 |
| 3 | O94806\|KPCD3_HUMAN | Serine/threonine-protein kinase D3 OS=Homo sapiens GN=PRKD3 PE=1 SV=1 | 3338 |
| 4 | P0DMV8\|HS71A_HUMAN | Heat shock 70 kDa protein 1A OS=Homo sapiens GN=HSPA1A PE=1 SV=1 | 1858 |
| 5 | P34931\|HS71L_HUMAN | Heat shock 70 kDa protein 1-like OS=Homo sapiens GN=HSPA1L PE=1 SV=2 | 840 |
| 6 | P11142\|HSP7C_HUMAN | Heat shock cognate 71 kDa protein OS=Homo sapiens GN=HSPA8 PE=1 SV=1 | 782 |
| 7 | P07437\|TBB5_HUMAN | Tubulin beta chain OS=Homo sapiens GN=TUBB PE=1 SV=2 | 626 |
| 8 | P68371\|TBB4B_HUMAN | Tubulin beta-4B chain OS=Homo sapiens GN=TUBB4B PE=1 SV=1 | 593 |
| 9 | Q9BVA1\|TBB2B_HUMAN | Tubulin beta-2B chain OS=Homo sapiens GN=TUBB2B PE=1 SV=1 | 500 |
| 10 | P68363\|TBA1B_HUMAN | Tubulin alpha-1B chain OS=Homo sapiens GN=TUBA1B PE=1 SV=1 | 486 |
| 11 | P54652\|HSP72_HUMAN | Heat shock-related 70 kDa protein 2 OS=Homo sapiens GN=HSPA2 PE=1 SV=1 | 348 |
| 12 | P11021\|GRP78_HUMAN | 78 kDa glucose-regulated protein OS=Homo sapiens GN=HSPA5 PE=1 SV=2 | 314 |
| 13 | P38646\|GRP75_HUMAN | Stress-70 protein, mitochondrial OS=Homo sapiens GN=HSPA9 PE=1 SV=2 | 264 |
| 14 | P62913\|RL11_HUMAN | 60S ribosomal protein L11 OS=Homo sapiens GN=RPL11 PE=1 SV=2 | 213 |
| 15 | Q5VTE0\|EF1A3_HUMAN | Putative elongation factor 1-alpha-like 3 OS=Homo sapiens GN=EEF1A1P5 PE=5 SV=1 | 148 |
| 16 | P08238\|HS90B_HUMAN | Heat shock protein HSP 90-beta OS=Homo sapiens GN=HSP90AB1 PE=1 SV=4 | 114 |
| 17 | P23396\|RS3_HUMAN | 40S ribosomal protein S3 OS=Homo sapiens GN=RPS3 PE=1 SV=2 | 106 |
| 18 | O75688\|PPM1B_HUMAN | Protein phosphatase 1B OS=Homo sapiens GN=PPM1B PE=1 SV=1 | 84 |
| 19 | Q06830\|PRDX1_HUMAN | Peroxiredoxin-1 OS=Homo sapiens GN=PRDX1 PE=1 SV=1 | 81 |
| 20 | P07900\|HS90A_HUMAN | Heat shock protein HSP 90-alpha OS=Homo sapiens GN=HSP90AA1 PE=1 SV=5 | 80 |
| 21 | P31943\|HNRH1_HUMAN | Heterogeneous nuclear ribonucleoprotein H OS=Homo sapiens GN=HNRNPH1 PE=1 SV=4 | 77 |
| 22 | P63173\|RL38_HUMAN | 60S ribosomal protein L38 OS=Homo sapiens GN=RPL38 PE=1 SV=2 | 74 |
| 23 | P62249\|RS16_HUMAN | 40S ribosomal protein S16 OS=Homo sapiens GN=RPS16 PE=1 SV=2 | 67 |
| 24 | P02768\|ALBU_HUMAN | Serum albumin OS=Homo sapiens GN=ALB PE=1 SV=2 | 64 |
| 25 | Q9H4B7\|TBB1_HUMAN | Tubulin beta-1 chain OS=Homo sapiens GN=TUBB1 PE=1 SV=1 | 61 |
| 26 | Q9H3K6\|BOLA2_HUMAN | BolA-like protein 2 OS=Homo sapiens GN=BOLA2 PE=1 SV=1 | 59 |
| 27 | P27635\|RL10_HUMAN | 60S ribosomal protein L10 OS=Homo sapiens GN=RPL10 PE=1 SV=4 | 59 |
| 28 | P05141\|ADT2_HUMAN | ADP/ATP translocase 2 OS=Homo sapiens GN=SLC25A5 PE=1 SV=7 | 57 |
| 29 | P13639\|EF2_HUMAN | Elongation factor 2 OS=Homo sapiens GN=EEF2 PE=1 SV=4 | 52 |
| 30 | P27348\|1433T_HUMAN | 14-3-3 protein theta OS=Homo sapiens GN=YWHAQ PE=1 SV=1 | 51 |
| 31 | Q15208\|STK38_HUMAN | Serine/threonine-protein kinase 38 OS=Homo sapiens GN=STK38 PE=1 SV=1 | 44 |
| 32 | P49411\|EFTU_HUMAN | Elongation factor Tu, mitochondrial OS=Homo sapiens GN=TUFM PE=1 SV=2 | 40 |
| 33 | P69905\|HBA_HUMAN | Hemoglobin subunit alpha OS=Homo sapiens GN=HBA1 PE=1 SV=2 | 32 |
| 34 | Q15365\|PCBP1_HUMAN | Poly(rC)-binding protein 1 OS=Homo sapiens GN=PCBP1 PE=1 SV=2 | 30 |
| 35 | P60866\|RS20_HUMAN | 40S ribosomal protein S20 OS=Homo sapiens GN=RPS20 PE=1 SV=1 | 28 |
| 36 | Q14568\|HS902_HUMAN | Heat shock protein HSP 90-alpha A2 OS=Homo sapiens GN=HSP90AA2P PE=1 SV=2 | 25 |
| 37 | Q9Y230\|RUVB2_HUMAN | RuvB-like 2 OS=Homo sapiens GN=RUVBL2 PE=1 SV=3 | 25 |
| 38 | P62857\|RS28_HUMAN | 40S ribosomal protein S28 OS=Homo sapiens GN=RPS28 PE=1 SV=1 | 25 |
| 39 | Q68CK6\|ACS2B_HUMAN | Acyl-coenzyme A synthetase ACSM2B, mitochondrial OS=Homo sapiens GN=ACSM2B PE=1 SV=2 | 25 |
| 40 | P60709\|ACTB_HUMAN | Actin, cytoplasmic 1 OS=Homo sapiens GN=ACTB PE=1 SV=1 | 25 |
| 41 | A5A3E0\|POTEF_HUMAN | POTE ankyrin domain family member F OS=Homo sapiens GN=POTEF PE=1 SV=2 | 25 |
| 42 | P62826\|RAN_HUMAN | GTP-binding nuclear protein Ran OS=Homo sapiens GN=RAN PE=1 SV=3 | 24 |
| 43 | Q14669\|TRIPC_HUMAN | E3 ubiquitin-protein ligase TRIP12 OS=Homo sapiens GN=TRIP12 PE=1 SV=1 | 24 |
| 44 | P62829\|RL23_HUMAN | 60S ribosomal protein L23 OS=Homo sapiens GN=RPL23 PE=1 SV=1 | 24 |
| 45 | P31947\|1433S_HUMAN | 14-3-3 protein sigma OS=Homo sapiens GN=SFN PE=1 SV=1 | 23 |
| 46 | Q03052\|PO3F1_HUMAN | POU domain, class 3, transcription factor 1 OS=Homo sapiens GN=POU3F1 PE=2 SV=3 | 22 |
| 47 | P49327\|FAS_HUMAN | Fatty acid synthase OS=Homo sapiens GN=FASN PE=1 SV=3 | 21 |
| 48 | P42677\|RS27_HUMAN | 40S ribosomal protein S27 OS=Homo sapiens GN=RPS27 PE=1 SV=3 | 20 |
| 49 | P83916\|CBX1_HUMAN | Chromobox protein homolog 1 OS=Homo sapiens GN=CBX1 PE=1 SV=1 | 20 |
| 50 | Q9Y265\|RUVB1_HUMAN | RuvB-like 1 OS=Homo sapiens GN=RUVBL1 PE=1 SV=1 | 19 |
| 51 | P46013\|KI67_HUMAN | Antigen KI-67 OS=Homo sapiens GN=MKI67 PE=1 SV=2 | 19 |
